# A happy place to be in: how beliefs about living places influence depression in Chinese international student returnees

**DOI:** 10.1186/s12889-024-21162-9

**Published:** 2024-12-27

**Authors:** Ruining Jin, Meiling Yin, Qiang Shen, Tam-Tri Le

**Affiliations:** 1 Civil, Commercial and Economic Law School, China University of Political Science and Law, Beijing, 100088 China; 2 China-EU School of Law, China University of Political Science and Law, Beijing, 100088 China; 3 Faculty of Materials and Manufacturing, Beijing University of Technology, Beijing, 100088 China; 4 Independent Researcher, Ho Chi Minh, 727300 Vietnam

**Keywords:** Chinese returnees, Mental health, Depression, International student, Happiness

## Abstract

**Background:**

The mental health of Chinese international student returnees is a critical concern impacting their well-being and successful reintegration into home society, especially in the post-COVID-19 era. This study examines how beliefs about changing living conditions, emigration intentions, and belief in fate influence depression levels among these returnees.

**Methods:**

A cross-sectional survey collected data from 1,014 returnees through WeChat public groups. Depression levels were measured using the Patient Health Questionnaire-9 (PHQ-9), and Bayesian analysis with Markov Chain Monte Carlo (MCMC) algorithms was employed for statistical examination.

**Findings:**

It was found that a stronger belief that changing one's living environment can bring happiness is associated with lower depression levels, highlighting the positive role of agency thinking in mental health. This association is moderated by emigration intentions and belief in fate. Specifically, among returnees who believe that changing living conditions enhances happiness, those with stronger intentions to emigrate from China exhibited lower depression levels, while those with a strong belief in fate showed higher depression levels.

**Implications:**

These results suggest that both agency and pathway thinking significantly affect the mental health of returnees. To support their well-being and reintegration, we recommend that policymakers create more diverse and inclusive environments within China that accommodate returnees' aspirations. Educational institutions should offer counseling services that bolster returnees' proactive strategies for achieving personal goals. Additionally, mental health interventions should address cultural beliefs, such as fatalism, which might hinder proactive coping mechanisms.

## Introduction

### Mental health issues among Chinese international student returnees

There is a lack of research on mental health issues among international students [1]. Out of more than 6,000 empirical publications published between 1980 and 2014 in nine journals commonly utilized by counseling psychologists, only 1.37% of studies focused on international students [1]. In the limited studies [2], found that 44% of the international graduate students at a university in the western United States reported experiencing emotional or stress-related issues that had a substantial negative impact on their overall well-being and academic performance. Some Asian international students even expressed greater levels of worry around self-harm, thoughts of suicide, and actual suicide attempts [3].

Chinese international student returnees’ mental health condition is a topic that deserves further examination. Most of the prior studies focused on Chinese international students’ mental health, particularly depression and anxiety in the West [4–8]. However, those who opted to go back to China might also face ongoing mental health problems. One recent study suggested that 47.9% of Chinese international student returnees sampled in that study suffered from a moderate level of depression in the post COVID-19 era [9]. For one thing, some of them experienced an involuntary displacement from the host nation to China during COVID-19 [10]. Prior research has suggested that involuntary migration can result in psychological stress among migrants [11, 12]. Furthermore, their return was accompanied by heightened Sino-US bilateral tension and the rise of cybernationalism in China [13, 14], therefore in this context, individuals who had an individualistic value pursuit or those who showed defiance toward quarantine preventative measures faced social stigmatization and discrimination [15]. In the post COVID-19 era, as China’s economic momentum slowed down, a chain of negative consequences occurred, such as brain drain and manufacturing desolation [16, 17]. As a result, domestic consumers’ confidence faded [18], which in turn, might bring about uncertainties and mental distress toward future careers. Also, the slowed economic growth may further deteriorate the exploitative workplace culture in developing countries (China’s current weekly working hours average at 48 [19]), resulting in further mental health issues among returnees seeking work-life balance [9, 20].

While prior studies have focused on returnees emigration intent [21], identity conflicts [22], place identity [23], and academic struggles [20], few focused on mental health issues from a cognitive perspective to probe how different perceptions such as residency, emigration intent, and belief in fate would alter mental health outcomes in a holistic manner.

### Hope as a buffer against mental distress

Hope can be one of the cognitive factors that holistically alters individuals’ level of mental distress when they face hardships. Through a psychological lens, Hope Theory can be a good theoretical framework to explain how hope shapes one’s mental health outcomes. Coined by Snyder, Hope Theory conceptualizes hope as the subjective belief in one's ability to devise strategies to attain desired objectives and to motivate oneself to implement those strategies [24, 25]. The concept consists of two primary elements: agency, which refers to the drive to pursue objectives, and pathways, which involve the strategic planning required to accomplish those objectives. A number of research has substantiated that hope is a crucial psychological attribute, acting as a safeguard against negative life experiences to lower mental distress [26–28]. However, prior studies are inconsistent on the impacts of the two elements when predicting the level of hope and behaviors. [25] deemed that agency and pathway thinking work in pair to sustain the overall level of hope, and when one is missing, the overall hope would be diminished. However, [29]’s study suggested otherwise, arguing that hope is based more on agency thinking. Similarly, [30]’s study offered similar findings, suggesting that agency thinking is more important than pathway thinking in the prediction of goal pursuit and performance. Behavior prediction-wise, [31]’s study suggested that strong agency thinking would predict persistence, while low pathway thinking is associated with a greater level of dropout during therapy.

### Agency-thinking from the perspective of place identity

From an identity perspective, Chinese international student returnees’ transnational mobility and living experience will bring about flexible identities upon them [32], including place identity–a sub-identity of transnational identity developed based on individuals’ long-time interactions with both natural and social environments at home and abroad [33, 34]. The formation of place identity would connect individuals with the collective norms, values, and characteristics of a place [35]. Therefore, one’s agency thinking can be reflected by the place identity he/she maintains. When confronted with challenges from the new environment, international students with a strong sense of place identity (agency thinking) might rely on their environmental past [34], which includes past experiences and memories, to preserve their connection to the place and offer a sense of hope to go back to the place they feel attached to when facing stressful situations [36, 37]. This agency thinking of place identity and attachment might include both a pro-home nation place identity [23], as well as a pro-host-nation place identity [38].

### Pathway thinking from the perspective of emigration intent and fate

In the context of Hope Theory, pathway thinking is critical for individuals to navigate challenges by identifying and conceptualizing pathways to achieve their goals [25]. For Chinese international student returnees facing socio-economic and cultural challenges upon returning home, the ability to generate effective pathways is essential for maintaining psychological well-being.

Emigration intent, whether it involves the intention to stay in China or to leave for another country, is a manifestation of pathway thinking. On the one hand, returnees who intend to remain in China could engage in pathway thinking by identifying strategies to improve their life satisfaction within their home country, such as leveraging their international experience to pursue career opportunities, foster personal growth, or contribute to societal development domestically [22, 39]. On the other hand, returnees who plan to emigrate again are also exercising pathway thinking. They perceive relocating abroad as a viable strategy to achieve their desired outcomes, such as better career prospects, a preferred lifestyle, or alignment with personal values [21, 22].

Meanwhile, belief in fate may influence pathway thinking by affecting an individual's perceived control over their life circumstances. Individuals with a strong belief in fate may attribute outcomes to external forces beyond their control [40–42]. This perception can hinder their ability to generate effective pathways because they may feel that personal efforts are inconsequential. In this scenario, whether a returnee intends to stay or leave, a strong belief in fate might reduce their proactive engagement in pathway thinking. For those intending to stay, they may feel resigned to their current conditions without actively seeking improvement. For those intending to leave, they might doubt the effectiveness of emigration as a means to achieve happiness, potentially leading to increased psychological distress.

### Current study

Given the trend of global talent mobilization, it is foreseeable that more and more international student returnees will go back to their home country to seek family reunions and career development opportunities. While prior studies have touched upon international student returnees reacculturation from the perspective of place identity [23], identity clusters and emigration intent [21], and locus of control [41, 43], limited studies have been conducted to present the mental well-being issue of returnees through the lens a comprehensive lens of hope to connect all the above. Therefore, to this end, this current study aims to bridge the gap by examining Chinese international student returnees’ depression levels associated with agency thinking and pathway thinking. Specifically, the current study has three research objectives:

1. To probe if the belief that changes in living conditions can bring happiness and fulfillment (agency thinking) may affect their depression level.
2. To investigate if and how their emigration intent (pathway thinking) would moderate this association.
3. To investigate if and how their trust in fate (pathway thinking) would moderate this association.

## Methodology

### Materials and variables

The survey consists of a total of 1014 Chinese international student returnees. It was conducted online through WeChat public groups. The researchers obtained entry to these groups by searching “returnee” as a keyword in the WeChat Public Account search. Upon subscribing to the public accounts, the researchers' status as returnees was confirmed via corresponding files and documents. Then researchers distributed informed consent forms and the aims of the study and provided the link to the online survey. In order to safeguard the safety and security of all participants, the names of the public groups included in the study will not be revealed, given the sensitive nature of the research. The groups’ sizes ranged from 200 to 500 users, with two groups consisting of US-based returnees, two groups consisting of UK-based returnees, and one group consisting of Australia-based returnees. The survey's data collection spanned from October 8, 2023, to January 30, 2024. Given that the new “brain drain” of China in recent years might include some returnees [44–46], and also because individuals experiencing culture shock and reverse culture shock often go through a temporary "honeymoon" phase [47], which can temporarily reduce the intensity of acculturative stress, the following inclusion criteria were listed: 1) participants must be born and raised in China and traveled abroad for educational reasons. 2) Participants must have returned and stayed in (or had stayed before leaving) China for at least one year after returning. 3) Participants have not participated in the same survey in any other WeChat public groups.

The survey questions were answered through the WeChat MiniApp *SurveyStar*. After a few rounds of screenings, including eliminating single responses for all questions and short-time answers (completed within 60 s), the final valid sample comprised 1014 participants. Among the 1014 participants, 455 responses were provided by male participants, accounting for approximately 44.87% of the total. There is a total of 523 female responses, which represents approximately 51.58% of the total. In addition, 36 respondents, accounting for approximately 3.55% of the total, defined themselves as "Others" within this group. Among the 1014 participants, 61.74% (626 participants) were between the ages of 18 and 30, 28.80% (292 participants) were between the ages of 31 and 40, and 9.47% (96 participants) were between the ages of 41 and 50. The studies involving human participants were reviewed and approved by the Institutional Review Board at China University of Political Science and Law, and relevant survey procedures were in line with the requirements of the Declaration of Helsinki. Written informed consent for participation in this study was provided by the participants. The data used in the study can be found at https://osf.io/vz425/.

Table 1 presents the variables.

**Table 1:** Variable description

Variable name	Meaning	Value
*Depression*	The participant’s total PHQ-9 score	Ranging from 0 to 27
*Emigrate*	The degree of the participant’s intention to emigrate	1. Definitely stay 2. Likely stay 3. Unsure 4. Likely migrate 5. Definitely migrate
*Change*	Participant’s response to the question “How much do you think that changes in living conditions/environments can bring happiness or fulfillment?”	1. No impact 2. Slight impact 3. Moderate impact 4. Strong impact 5. Extremely strong impact
*Destiny*	Participants’ response to the question “How much do you believe in fate/destiny?”	1. No belief 2. Slight belief 3. Moderate belief 4. Strong belief 5. Extremely strong belief

^*^All participants in this sample are over 18 years old

The variable *Depression* is a numeric variable with its value being the participant’s total score (ranging from 0–27) on the Patient Health Questionnaire-9 (PHQ-9) diagnosis [48]. The PHQ-9 is a self-report tool designed to assess the severity of depression. It consists of nine questions that correspond to the diagnostic criteria for major depressive disorder in the *Diagnostic and Statistical Manual of Mental Disorders* (DSM). PHQ-9 has been widely used in various psychological studies regarding depression among immigrants, international students, and other vulnerable groups’ mental health studies [9, 49, 50]. The variable *emigrate* represents the participant’s intention to emigrate, where “1” means definitely stay, and “5” means definitely emigrate. The variable *Change* refers to participants agreement to the possible impact of changing living conditions and environment on participants’ life satisfaction and happiness. The variable *Destiny* measures participants belief in fate/destiny, where “1” means “no belief” and “5” means “extremely strong belief”.

### Analysis procedure

Bayesian analysis aided by Markov Chain Monte Carlo (MCMC) algorithms was used in the current study. All model construction, analysis procedure, and result presentation are in alignment with the protocols of MCMC-aided Bayesian analytics for social sciences [51, 52]. The formula of the analytical model is as follows.

1 $$Depression \sim normal\left(\mu ,\sigma \right)$$

2 $${\mu }_{i}={\beta }_{0}+{\beta }_{Change}*{Change}_{i}+{\beta }_{Change*Emigrate}*{Change}_{i}*{Emigrate}_{i}+{\beta }_{Change*Destiny}*{Change}_{i}*{Destiny}_{i}$$

Regarding the outcome variable $Depression$, ${\mu }_{i}$ refers to participant $i$’s degree of depression level reflected by PHQ-9 scores. Participant $i$’s agreement to the impact of changing living conditions and environment on life satisfaction and happiness is ${Change}_{i}$. Participant $i$’s emigration intention is ${Emigrate}_{i}$. Participant $i$’s belief in fate is ${Destiny}_{i}$. The model has an intercept ${\beta }_{0}$ and coefficients ${\beta }_{Change}$, ${\beta }_{Change*Emigrate}$ and ${\beta }_{Change*Destiny}$.

**Fig. 1:**
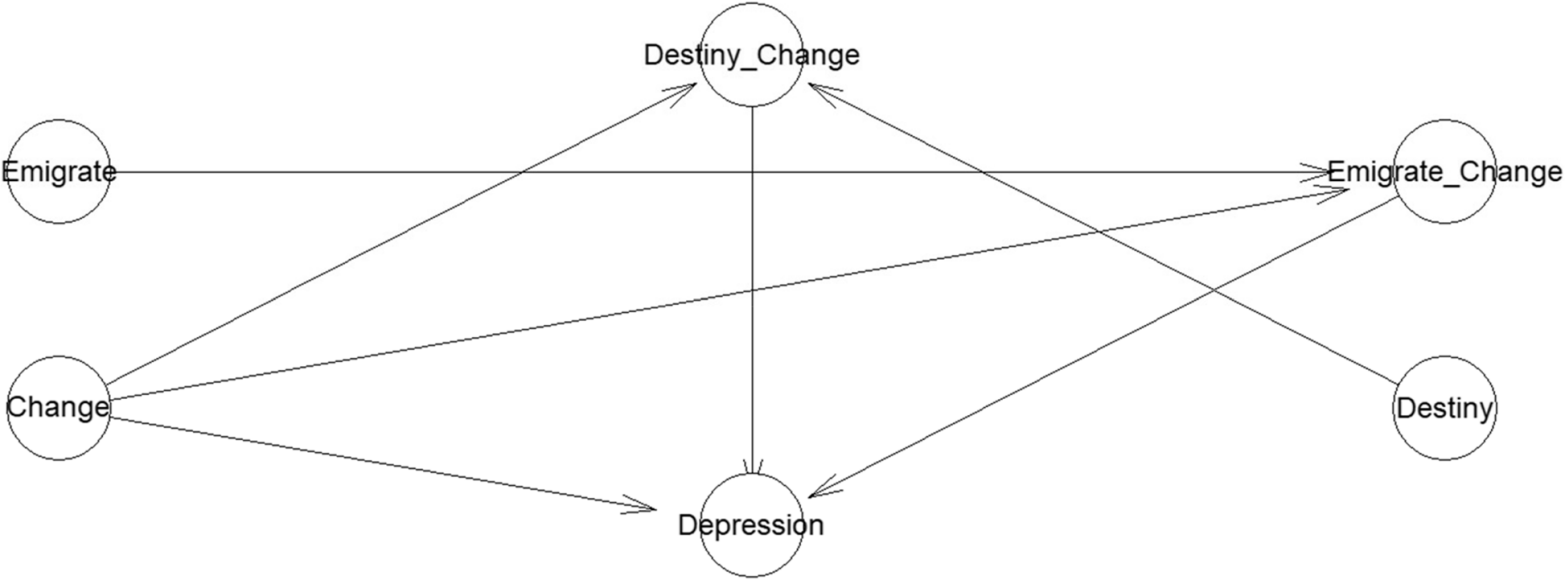
The model’s logical network

The model’s logical network is shown in Fig. 1.

Bayesian analysis aided by MCMC is statistically advantageous for providing high inference accuracy in exploratory analyses, as MCMC can function to minimize values’ inherent skewness in a sample. Chinese international student returnee population is a unique social group, which increases the difficulty of ensuring a very large sample size. MCMC algorithms can generate a large number of simulated data points based on the original data, increasing the number of effective data points in categories with low observations, and thus enhancing the predictive power of the model’s estimated posterior results.

The Bayesian approach views all characteristics probabilistically. Results are interpreted for parameters using the highest probability of occurrence in their posterior distributions, which helps improve the flexibility and accuracy of data interpretation in psychological studies [53–56]. Markov chain convergence’s evaluation is based on analysis of effective sample size (*n_eff*) and the Gelman-Rubin shrink factor (*Rhat*). The *n_eff* values should be more than 1000 for reliable inferences (McElreath, 2020), and the *Rhat* values should be 1 to indicate achieved Markov properties [57, 58]. The analysis was conducted with the use of the *bayesvl* package in R [59]. Markov chain convergence was also verified visually through trace plots, Gelman-Rubin-Brooks plots, and autocorrelation plots. The MCMC configuration is made up of 5000 total iterations, with 2000 warm-up iterations and 4 chains.

## Results

**Fig. 2:**
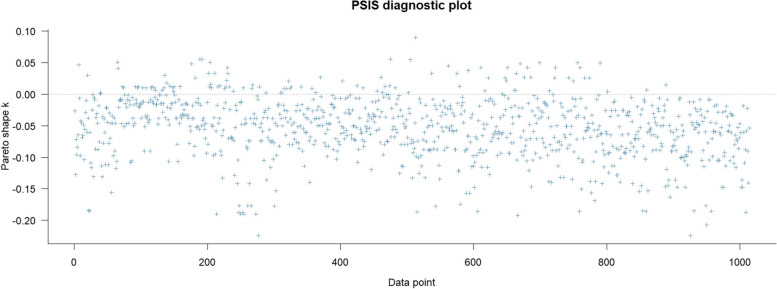
PSIS diagnostic plot

The PSIS diagnosis result is shown below (See Fig. 2). All *k* values are lower than the 0.5, indicating a healthy goodness-of-fit.

**Fig. 3:**
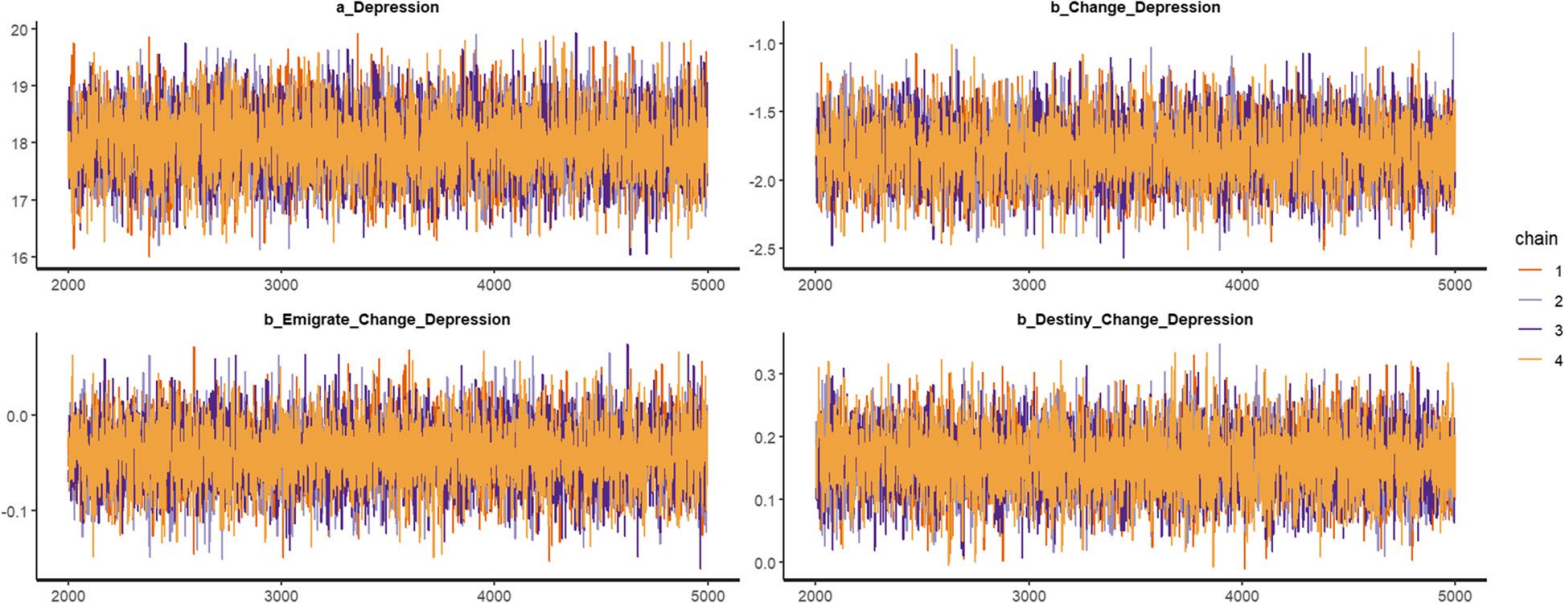
Trace plots

Posterior results are presented in Table 2. The statistical analysis shows good convergence of the model's Markov chains, given that the effective sample size (*n_eff*) is over 1000, and the Gelman-Rubin shrink factor (*Rhat*) is 1. These two values demonstrated that the posterior coefficients are reliable. Figure 3 is the trace plots where the colored lines are representing the Markov chains. It is observable that there is a fluctuation of lines around a central equilibrium following the warmup period, thus it could be seen as an indication of well-mixing and stationary qualities.

**Table 2:** Simulated posteriors

Parameters	Mean (M)	Standard deviation (S)	*n_eff*	*Rhat*
Constant	17.99	0.59	7776	1
*Chage*	−1.78	0.22	6400	1
Emigrate*Change	−0.04	0.03	9156	1
Destiny*Change	0.16	0.05	8766	1

**Fig. 4:**
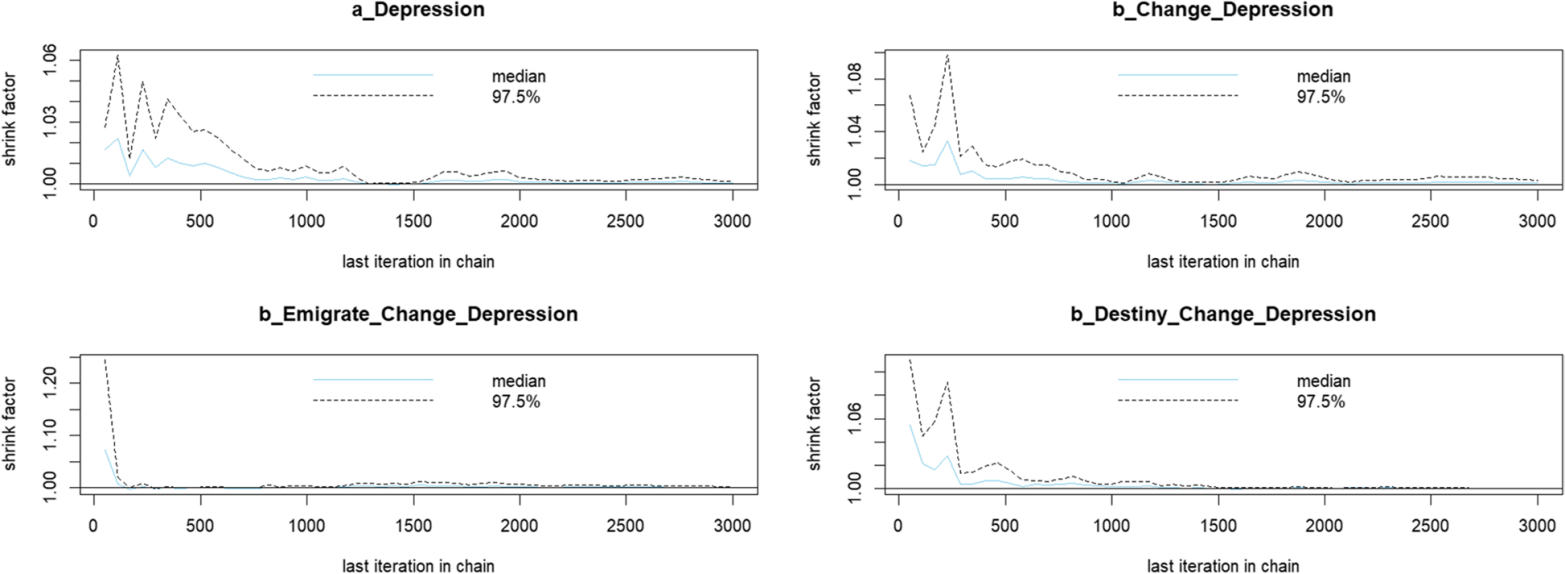
Gelman-Rubin-Brooks plots

**Fig. 5:**
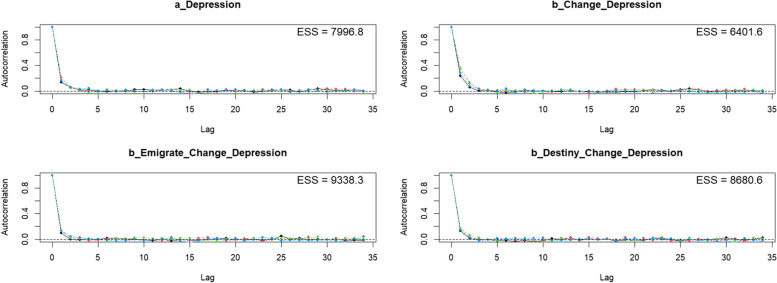
Autocorrelation plots

The Gelman-Rubin-Brooks plots (Fig. 4) shows that *Rhat* values drop quickly to 1 during the warm-up period. Furthermore, the autocorrelation plots (Fig. 5) illustrate a fast elimination of problematic autocorrelation in simulated data points during the MCMC processes.

Based on the analysis results (See Table 2), there is a clear negative association between returnees’ belief that the change of their current environment will bring life satisfaction and happiness and their depression level (${M}_{Change}$ = −1.78 and ${S}_{Change}$= 0.22). This association is moderated by both migration intentions and belief in destiny. Specifically, the intention to emigrate from China exerts a negative moderating effect ${(M}_{Emigrate*Change}$= −0.04 and ${S}_{Emigrate*Change}$ = 0.03). Conversely, belief in destiny suggests a positive moderating influence on this association ${(M}_{\text{Destiny}*\text{Change}}$= 0.16 and ${S}_{\text{Destiny}*\text{Change}}$ = 0.06).

**Fig. 6:**
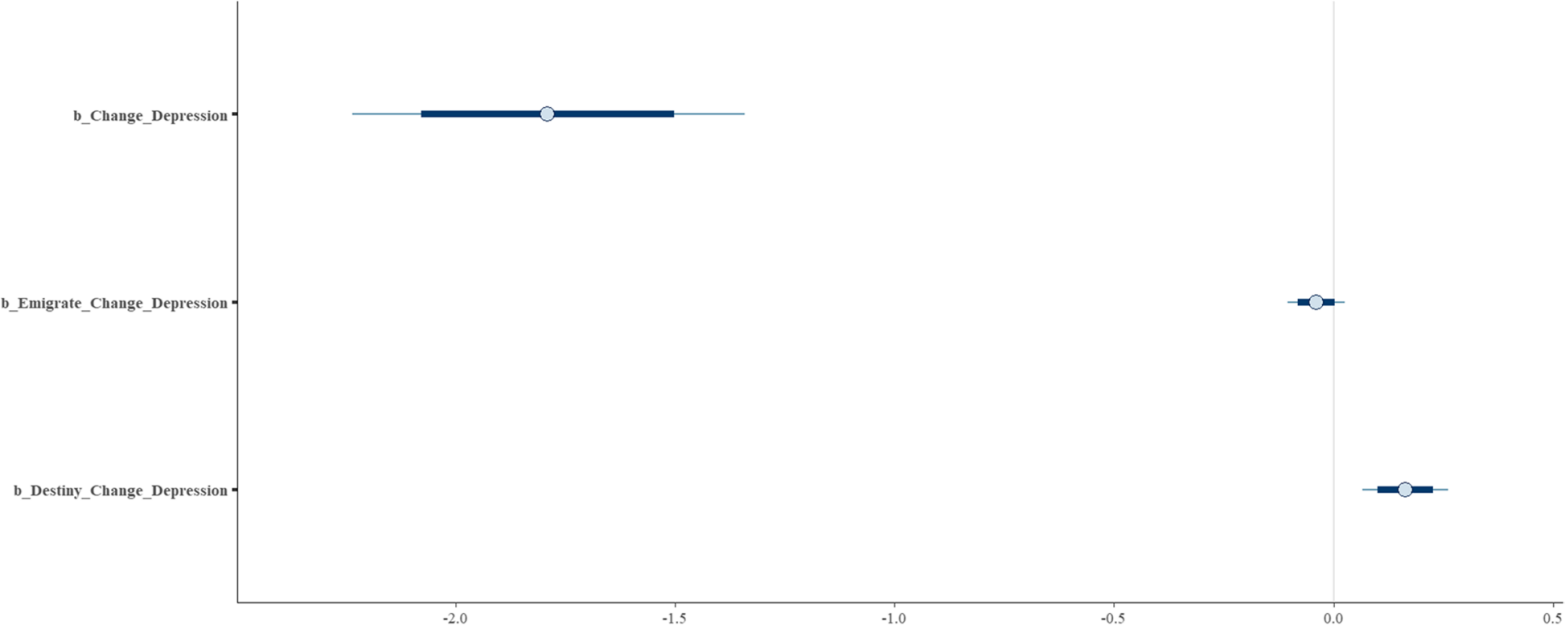
Posterior distributions

In Fig. 6, we can observe that the posterior distributions of *Change* are entirely on the negative side and Emigrate*Change is almost entirely on the negative side, too, whereas Destiny*Change are entirely on the positive side.

## Discussions

Using Bayesian analysis aided by MCMC algorithms on 1014 Chinese international student returnees, the current study found a negative association between the perception that altering their current living environment will lead to their life satisfaction and returnees’ depression level. In addition, this association was moderated by both their emigration intention and belief in destiny. Specifically, among returnees who believed more strongly that changes in living conditions can bring happiness, those with a stronger intention to emigrate away from China had a lower depression likelihood, whereas those with a strong belief in destiny had a higher depression likelihood**.**

### Discussion of RQ1

The finding that returnees’ belief in altering their current environment to achieve happiness is associated with lower depression levels is consistent with Hope Theory, which emphasizes that agency thinking can independently enhance mental health by empowering individuals to navigate stressful situations [29]. Having experienced diverse cultural environments in both China and the West, Chinese international student returnees have likely explored various places and formed diverse place identities. When these returnees believe that changing their environment will bring happiness, this reflects their agency thinking, namely the confidence and motivation to pursue desired outcomes through proactive change. This agency thinking can reflect their place identity, as they are committing to a specific place that aligns with their personal values and aspirations, regardless of whether it is pro-home nation or pro-host nation. Thus, the place-identity-led agency thinking would increase their hope, which in turn safeguard returnees from negative experiences during reacculturation. Such a finding is consistent with prior studies on hope and college students’ mental health outcomes [60–62]. This agency thinking and solidification of place identity also align with the concept of identity achievement status [63, 64], which involves active exploration and commitment to certain values and roles, resulting in a coherent sense of self. Studies have also demonstrated that individuals with identity achievement status exhibit better psychological functioning and mental health [9, 65–67].

Conversely, those who lack strong agency thinking and place identity, or who do not believe that changing their living conditions would bring fulfillment, may have a lower level of hope. This diminished hope can render them more vulnerable to sociocultural conflicts and socioeconomic setbacks during reintegration, therefore increasing the risk of depression and other mental health issues. This finding is also supported by prior studies on lack of agency thinking and the associated mental health problems [30, 68].

### Discussion of RQ2

The findings revealed that among returnees who strongly believed that altering their environment could bring happiness, those with a stronger intention to emigrate from China exhibited lower levels of depression. This moderating effect of emigration intentions is intuitive for returnees with a strong pro-host-nation place identity. After all, strong emigration intent offers them a strong pathway thinking in addition to their agency thinking discussed in RQ1 to pursue happiness and fulfillment in the host nation they feel attached to.

Nonetheless, this finding revealed a more nuanced and somewhat counterintuitive pattern: it seems paradoxical that returnees with a pro-home nation place identity (those who think changing places to live in China could offer happiness and fulfillment) would contemplate emigration. However, the current socioeconomic and sociopolitical landscape in China might be mentally challenging for all returnees. Namely, surging domestic nationalism during and after the COVID-19 might form outgroup bias, leading to stigmatization and discrimination towards returnees who have a sense of transnational identity [14, 15]. In addition, COVID-19’s impact on the world economy and the Chinese government’s preventive regulations also stifled China’s economic growth trajectories, reflected by the decoupling between China and the West, the desolating manufacturing [16, 17], fading domestic consumer confidence [18], and an exploitative workplace culture [19, 20]. Therefore, given the domestic sociopolitical and sociocultural landscape, it is reasonable to argue that although returnees with a pro-China place identity maintain strong motivation (agency thinking) to find happiness and fulfillment in China, economic hardships, socio-cultural dissonance, employment difficulties, and other systemic challenges would hinder their ability to set viable strategies (pathway thinking) to achieve the goals of finding happiness and fulfillment domestically [9, 20, 22]. This finding is also consistent with prior studies, which indicated that when individuals possess strong agency but low pathway thinking, they may experience frustration and are more likely to disengage from their initial goals and drop out [30, 31]. Therefore, emigration intent, in this regard can offer them a pathway thinking to seek happiness and fulfillment overseas such as in the host nation, and this pathway thinking can work independently to improve their hope and lower their depression level, even though returnees with a pro-home nation place identity might lack agency thinking [30, 31].

### Discussion of RQ3

A strong belief in destiny had a positive moderating impact on the association in RQ1, meaning that a strong belief in fate would result in higher depression levels among those who believe that changing their environment can enhance happiness. This result can also be quite interesting. A strong belief in fate or destiny can affect pathway thinking by fostering an external locus of control, wherein individuals perceive that outcomes are determined by external forces beyond their control [69]. This perception can undermine their ability to identify and pursue effective strategies, as they may feel that personal efforts are inconsequential in influencing their circumstances. Prior studies have linked an external locus of control to poorer mental health outcomes, as individuals with an external locus of control are more likely to experience depression and anxiety due to feelings of helplessness and lack of control over their lives [40, 42].

In the case of Chinese international student returnees who believe that changing their living conditions can bring happiness (reflecting strong agency thinking), a strong belief in fate may conflict with their ability to generate viable pathways to achieve this goal. The influence of fate in Chinese culture is deeply rooted in Confucianism, which emphasizes role ethics and social harmony [70]. Confucian philosophy advocates for individuals to fulfill their prescribed social roles and duties, often prioritizing collective well-being over personal aspirations [71, 72]. Returnees who hold a strong belief in fate may feel obligated to adhere to traditional expectations, such as filial piety, which can conflict with their personal goals of changing their living conditions to seek happiness. For example, a female returnee who believes in fate may feel compelled to accept her role as a daughter, fulfilling duties like caring for her parents. While she may possess the motivation to pursue personal happiness (agency thinking), her belief in fate and associated cultural obligations hinder her ability to generate and act upon pathways that involve altering her living conditions, such as emigrating from China and pursuing career opportunities abroad. This internal conflict between personal desires and perceived destiny can exacerbate feelings of frustration and helplessness, therefore increasing depression levels.

### Implications

The findings from this study provide several important implications for policymakers, educators, mental health professionals, and future research.

First, the positive association between agency thinking and lower depression levels suggests that empowering returnees to exercise agency can enhance their mental well-being. Policymakers should create supportive environments that enable returnees to pursue their personal and professional goals within China. This could involve providing career development opportunities tailored to returnees, recognizing and leveraging their international experience, and fostering inclusive communities that accommodate diverse aspirations. By facilitating conditions where returnees feel they can effect meaningful change in their lives, policymakers can help reduce depression levels in this population. The city of Guangzhou is a great example, per [23], which is actively participating in the process of globalization and serves as an intellectual hub attracting individuals who have returned to their home country with its favorable policies and its enjoyable living conditions.

Secondly, policies should not restrict mobility but rather provide flexible opportunities both domestically and internationally. Policymakers could facilitate international collaborations, ease visa restrictions, and consider policies that support dual citizenship or multiple residencies. Such measures would allow returnees to navigate between home and abroad more freely, enhancing their pathway thinking and contributing to better mental health outcomes. Chinese international student returnees who have extensive experience living in foreign countries, play a crucial role in promoting mutual understanding between Eastern and Western cultures. They are also associated with enhanced creativity, problem-solving skills, and professional success in multicultural environments [73]. Therefore, offering meaningful alternatives for lifestyles and respecting individual differences are in accordance with China's national interests.

Thirdly, educational institutions should incorporate modern psychological concepts into curricula that promote an internal locus of control and encourage proactive problem-solving skills. While respecting traditional values, educators can balance them with teachings that empower individuals to take charge of their destinies. Mental health professionals should adopt culturally sensitive approaches that help returnees reconcile traditional beliefs with strategies that enhance agency and pathway thinking.

Future research should explore longitudinal effects of these cognitive factors on mental health, investigating how changes in beliefs about living conditions, emigration intentions, and fate influence depression over time. Such studies could provide deeper insights into the dynamics of returnees' mental health and inform the development of targeted interventions.

### Limitations

The present study has some limitations. First, the reliance on self-reported data may introduce response biases, including social desirability and recall bias, leading participants to underreport or overreport their mental health conditions due to personal or cultural factors. Additionally, the cross-sectional design limits the ability to infer firm causality between variables, necessitating longitudinal studies to confirm suggested causal relationships and track changes over time. Furthermore, conducting the survey online through WeChat may have excluded less tech-savvy returnees and impacted the depth and nuance of responses compared to pencil-and-paper survey.

## Data Availability

The data used in the study can be found at https://osf.io/vz425/.
